# Metals Biotribology and Oral Microbiota Biocorrosion Mechanisms

**DOI:** 10.3390/jfb14010014

**Published:** 2022-12-23

**Authors:** Nicola Contuzzi, Giuseppe Casalino, Antonio Boccaccio, Andrea Ballini, Ioannis Alexandros Charitos, Lucrezia Bottalico, Luigi Santacroce

**Affiliations:** 1 Department of Mechanics, Mathematics and Management, Polytechnic University of Bari, Via Orabona 4, 70125 Bari, Italy; 2 Department of Precision Medicine, University of Campania “Luigi Vanvitelli”, 80138 Naples, Italy; 3 Emergency/Urgent Department, National Poisoning Center, Riuniti University Hospital of Foggia, 71122 Foggia, Italy; 4 Interdepartmental Research Center for Pre-Latin, Latin and Oriental Rights and Culture Studies (CEDICLO), University of Bari, 70121 Bari, Italy; 5 Department of Interdisciplinary Medicine, Microbiology and Virology Unit, University of Bari “Aldo Moro”, 70126 Bari, Italy

**Keywords:** metal-based biomaterials, oral implants, toxicology, biotribology, microbiology, oral microbiota, immunity, biochemistry, biomedical applications

## Abstract

During the last decades, metal-based biomaterials have been extensively explored to be used as biocompatible metals for biomedical applications, owing to their superior mechanical properties and corrosion resistance. Consequently, for long-term implanted medical devices, to assure the biomaterials’ reliability, functionality, and biocompatibility, studying the various bio-tribological damage mechanisms to obtain the optimum properties is one of the most important goals. In this review, we consider the most important metal-based biomaterials such as stainless steel, alloys of titanium (Ti), cobalt-chromium (Co-Cr), and Nichel-Titatium (Ni-Ti), as well Magnesium (Mg) alloys and with Tantalum (Ta), emphasizing their characteristics, clinical applications, and deterioration over time. The influence of metal elements on biological safety, including significant effects of metal-based biomaterials in dentistry were discussed, considering the perspectives of surface, mechanical properties, corrosion behaviors, including interactions, bio-mechanisms with tissues, and oral environments. In addition, the role of the oral microbiota was explored due to its role in this erosion condition, in order to further understand the mechanism of metal-based biomaterials implanted on the microflora balance of aerobic and anaerobic bacteria in an oral environment.

## 1. Introduction 

**Figure 1:**
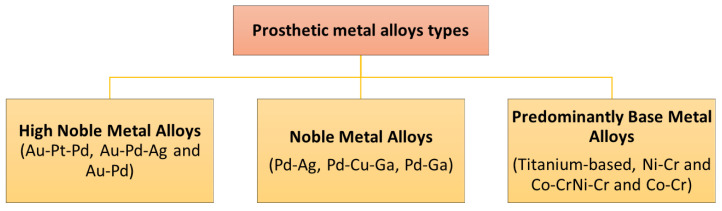
The metal oral implants and dental alloys for immobile and mobile prosthetic work, based on the percentage content by weight of noble metals (such as Au, Pd, Pt, Ag), in the three categories: (a) high noble metal alloys with a content percentage of noble metals greater than 60%, (b) noble metal alloys with a content of noble metals between 25–60%, and (c) predominantly, base metal alloys may also contain noble metals, in a percentage less than 25% by weight. The total percentage of Ni and Cr in Ni-Cr alloys and Co and Cr in Co-Cr alloys, respectively, cannot exceed 85%. Source: American Society for Testing and Material and ADA Standards Committee on Dental Products (SCDP).

Metal biomaterials have been used and continue to be used for both the restoration of partial dentition and in the surgical treatment of bone lesions. Thanks to their physical properties such as mechanical strength, machining, and shaping (i.e., casting, forging, ductility, etc.), which are relatively easy, together with electrical conductivity, have caused them to be the most usable [1]. These properties are due to the metallic bond that prevails in metals and to the mobile electrons they possess. One of the most important, if not the most important, properties of metallic biomaterials is their resistance to corrosion, since high corrosion resistance implies high biocompatibility and the retention of the biomaterial [2]. Hence, they were initially used in orthopedics and dentistry, due to their hardness and resistance to mechanical stress. Since access to the teeth was readily available and it was also possible to maintain good antiseptic conditions, the scientists monitored results. Today, they are widely used in maxillofacial surgery or orthopedic prostheses for the restoration of bone defects (support plates, screws, wires, hip heads, etc.), where they promote bone healing, correction of bone deficits (from accidents, cancer, or other) and the treatment of dentition [3]. However, since intense corrosive conditions mainly develop in the oral cavity due to eating habits, oral fluids, and local microbiota, the metal dental implants are very easily damaged. This has allowed the development of research in the field of dentistry. To design biomaterials for deformities, diseases, and injuries treatments, they must first understand the evolving nature and composition of ossified bone so that the biomaterial can respond to mechanical and hormonal stimuli. Bone is not a homogeneous material, similar to the metals used in most implants [4,5]. Human bone can match mechanical properties and strength (bone tensile strength can be almost equal to that of iron), however bone is three times lighter and ten times more flexible than iron. The plates for osteosynthesis, metal-ceramic crowns and bridges, and dental implants are typical cases of application of these biomaterials [6]. Internationally, coated or uncoated titanium alloys (Ti), nickel-chromium alloys (Ni-Cr), chromium-cobalt alloys (Cr-Co), and stainless steel are widely used in both prosthesis and its bone surgery [7]. Thus, the main components of the elemental composition of dental alloys can be based on Ag, Pd, Co, Cr, Mo, Fe, C, Si, Mn, Ni, Cr, Mo, Fe, C, Be, Mn, Pd, Ga, Cu, cpTi Ti, O, N, C, Fe, H, Ag, Au, Cu, In, Pd, Pt, and Zn (Figure 1) [8,9]. 

## 2. Characteristics and Toxicity of Metal Alloy Biomaterials 

Austenitic stainless-steel alloys are used almost exclusively as biomaterials, i.e., alloys with austenite (γ-Fe) as the main phase. The austenitic stainless steels high fatigue strength and high yield strength, especially when quenched, cause them to be a suitable material for the fabrication of implants located in areas with high mechanical loads. For maxillofacial surgery, steel is used for the manufacture of osteosynthesis devices (materials), i.e., of wires, plates, screws, etc. [10,11]. In orthodontics, stainless steel is used in the manufacture of orthodontic wires to move teeth. However, under conditions where mechanical stress and reduced oxygen concentration coexist, stainless steel corrodes rapidly, releasing metal ions (Cr, Fe, Ni) and particles in the body [12]. Bone responds to mechanical stimuli and reshapes itself according to the mechanical needs of the environment to maintain its integrity, unlike stainless steel. The mechanical loads exerted on the bone cause micro-deformations that rarely exceed 3%. In fact, the cells of the bone tissue receive and recognize the mechanical “signals” of the environment and bring about changes in bone mass and bone architecture [13]. The bone in the unloading area stops receiving the mechanical stimuli necessary for its homeostasis and is absorbed. This condition is called bone resorption due to discharge or osteoporosis due to disuse (stress shielding) [14]. Nevertheless, there is a critical limit of micro-deformations, which is necessary for bone homeostasis, and when their magnitude is below this critical limit (i.e., disuse and other) the bone is reabsorbed with the decrease in mass. The biomechanical incompatibility of the metal—bone biomaterial in terms of the elastic modulus considerably limits the transmission of mechanical loads from the much more “rigid” metal biomaterial to the more “flexible” adjacent bone [15]. The result of this condition is the loosening of the implant metal and the induction of refraction for osteopenia of the bone, when the osteosynthesis materials are removed. The increase in stainless-steel alloys’ corrosion resistance is achieved by coating with a thin passive layer of Cr_2_O_3_ that firmly adheres to their surface [16,17]. 

In dentistry, chromium-cobalt alloys have been used for the building of prosthetic structures, i.e., bridges and crowns. The composition of these alloys is usually Cr (about over 30%), Mo (over 7%), and they contain small quantities of Ni (<1%), which, as is well known, has shown an allergenic or carcinogenic effect [18,19]. They also contain traces of Mn, Fe, Si, N, and C. The presence of carbon in the alloy greatly increases its hardness because carbides (a compound composed of carbon and a metal) are formed. The alloys, depending on the content of the carbon, are distinguished in low content, when its concentration is about 0.05%, and high content, when the concentration is about 0.25%. The thermal expansion coefficients of these alloys are similar to those of ceramics. Cr-Co alloys have a higher thermal expansion coefficient than Ni-Cr [20]. In cobalt-chromium alloys, two phases coexist: the dominant Co mother phase and the carbide phase (mainly Cr carbides). The phase ratio and dispersion of the carbides affect the hardness and mechanical behavior of the alloy. High carbon Cr-Co-Mo alloys are the metal biomaterials with the highest abrasion resistance, which is attributed to the carbide phase. Since a stable passive film of Mo and Cr oxides forms on the surface of the alloy, they show good corrosion resistance, although there are reports of Cr^3+^ and Co^3+^ ions being released into fabrics due to their corrosion [21]. The method of processing the alloy also determines its mechanical behavior. The manufacture of dental prostheses is obtained either by casting with the “lost wax” technique or by mechanical processing, i.e., forging, turning, etc. or by powder metallurgy, or hot isostatic pressing. Mechanical processing and powder metallurgy improve mechanical behavior and show higher tensile strength, higher yield strength, and higher fatigue strength than the corresponding cast alloys [22]. 

**Figure 2:**
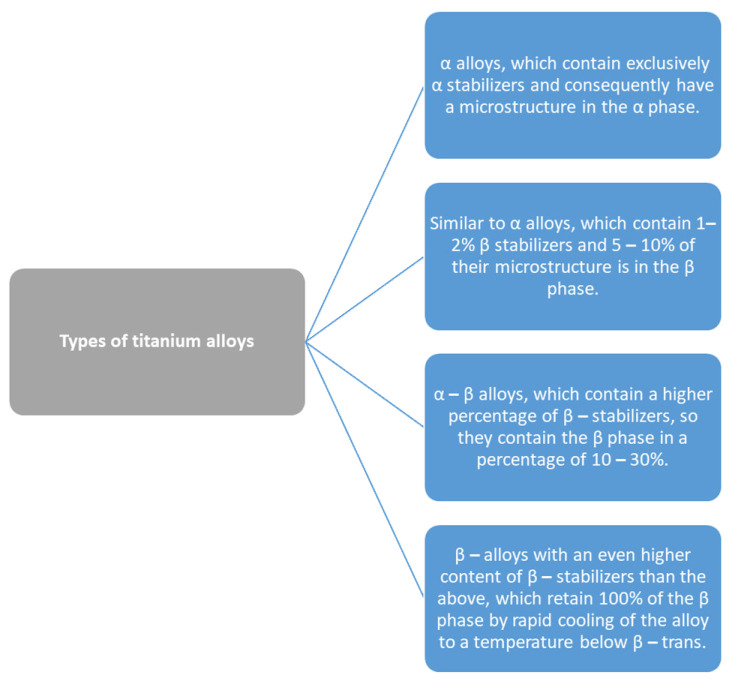
Types of titanium alloys are classified according to their metallurgical characteristics as follows. A-β titanium alloys (such as Ti–6Al–4V, Ti–6Al–7Nb, Ti–5Al2.5Fe) show a high resistance to corrosion and fatigue and find application in cases where a high mechanical load is required, such as ex. as osteosynthesis materials [29,30,31].

Titanium, useful in the manufacture of metal-ceramic works, has a low specific weight (4.5 g/cm^3^), a high resistance to corrosion, optimal mechanical properties as well as biocompatibility, and optimal radiopacity [23,24]. Hence, unalloyed Ti and its alloys are recognized as suitable to be optimum biomaterials [25]. However, even small variations in the O_2_ content significantly affect the mechanical behavior of titanium, because the O_2_ atoms scattered in the crystal lattice between the titanium atoms prevent the crystal planes from moving. Pure titanium alloys contain oxygen that, depending on the content, are divided into cpTi grade I, II, III, and IV [26]. The pure Ti grade IV shows the highest oxygen content (0.40%). The content of N, C, H, and Fe does not differ significantly between the four grades of commercially pure titanium. Iron is also added to titanium alloys to increase the corrosion resistance. Therefore, the small variations of the O_2_ content affect the plasticity of the titanium, which is reduced as its yield strength increases instead. Titanium, as a transition element, since it does not have the completed electron outer shell, easily forms solid solutions with various data. The alloying elements are divided into α-stabilizers, β-stabilizers, and “neutral” elements (Figure 2) [27,28]. 

The high biocompatibility with osseointegration and its high resistance to corrosion have caused it to be a biomaterial of choice in dentistry for the realization of dental implants, in maxillofacial surgery and more. The osseointegration (which would be the direct biomechanical connection, without the intervention of fibrous connective tissue, of the living bone tissue with the implant) would ensure a better functional stability [32]. This occurs for example in bone-integrated dental implants with titanium “artificial roots” in jaw bones, effective for the treatment of both partial and total dentition with a very high success rate of the implant, and it has been found that for the lower jaw it varies between 87 and 98% [33]. Finally, it is believed that chronic exposure can release Aluminum and Vanadium ions (due to corrosion) with toxic effects that can be a risk factor for the development of polyneuropathy, Alzheimer’s disease, and others. In fact, Vanadium can have a toxicity both in its elemental form, but also in the form of the oxide V_2_O_5_ [34].

Magnesium (*Mg*) is essential for bone and cellular metabolism, replaces calcium in hydroxyapatite, membrane function, and integrity and does not demonstrate toxicity (even at high concentrations) [35]. Under certain condition, the vulnerability of magnesium to corrosion is an advantage when it is controlled. Indeed, the biodegradation extent and rate of this biomaterial must be considered with the pace of tissue regeneration, so that when the regeneration is complete (the biomaterial must be completely degraded) [36]. This finds its application to second generation dental implants that are placed for permanent use together with a scaffold for tissue regeneration in the damaged area, only for a certain period until they are replaced by newly formed tissue. 

However, magnesium degrades too rapidly and produces a hydroxide layer on the surface. In the human environment, hydroxide is converted into highly soluble magnesium chloride that promotes the corrosion of the implant. Furthermore, hydrogen evolution during corrosion can cause the formation of gas bubbles that can cause the separation of the human tissue to the implant and slow down the healing process [37].

To increase the mechanical strength and the corrosion resistance of magnesium, the most common used metal is aluminum. It is believed that at low concentrations of aluminum a layer of Al_2_O_3_ forms, which protects the alloy and is less soluble than Mg (OH)_2_ [38]. However, aluminum belongs to the category of metals that promotes Alzheimer’s, so its use is avoided. The addition of rare earth elements that include the elements Lanthanum (La), Luthenium (Lu), Scandium (Sc), and Yttrium (Y) to Mg increases mechanical and corrosion resistance and their biological behavior has been studied in vitro by Shi et al. [39]. Recently, researchers have turned to non-toxic alloys (such as those with Ca, Mn, Zn, and Zr) that alter the mechanical properties and corrosion resistance of magnesium [40]. 

Manganese (Mn) added to magnesium alloys increases corrosion resistance but does not increase the mechanical strength of the alloy, which is why it is used in conjunction with Al and Zn. The Mn in high concentrations have toxic behavior. The Zn is an element that is often used to increase the mechanical properties of magnesium alloys and, in high concentrations, it behaves as toxic for organisms [41,42]. The bioabsorbable Mg-Zn alloy is marketed under the trade name ZE41 (Mg-4Zn-1RE). Zr is one of the first metals used in magnesium alloys. It increases the mechanical and corrosion resistance of the alloy, which is why it is used in dental materials [43,44]. The Mg-Li alloys as bioabsorbable dental implants have shown in vitro that the biodegradation and absorbability times of the alloys are equivalent to those required for bone regeneration [45]. 

Finally, Ni-Ti alloys are “intelligent” biomaterials because, in addition to their high resistance to corrosion (in particularly corrosive environments, such as the oral cavity), they combine elasticity, excellent formability (shape memory), and, therefore, biomimetic behavior [46,47]. 

Zinc (Zn)-based alloys are considered as a third-generation metallic material thanks to their biodegradable behavior, ideal corrosion rate, and acceptable biocompatibility [48]. Zinc plays an essential role in bone metabolism, as it stimulates osteoblast bone formation and inhibits osteoclast differentiation [49]. Furthermore, biodegradable Zn guarantees sufficient mechanical support and presents an appropriate biodegradation rate able to prevent inflammation. The addition of Zn increases resistance to corrosion also in adding and mixing alloys with Fe, Ni, and Cu. Li played a significant role in strengthening the Zn matrix, while the ductility declined significantly with Li and Mg additions.

In the corrosive environment, pure Zn is covered by an intact corrosion layer with tiny precipitates on the surface. Binary Zn alloys presented a corrosion morphology similar to that of pure Zn except Zn-0.8Ca alloy, where precipitates in a much larger size were observed [50].

Iron is a nontoxic and biodegradable metal. It is commonly used for medical applications due to its ease of manufacturing and mechanical reliability [51]. However, its degradation rate is quite slow. Alloying iron with Mn or Mn-Pd can help iron degrade faster and more uniformly [52]. However, the Fe-Mn alloy form an oxide layer between the tissue and implant, that can slow down the degradation rate of the implant [53]. 

In the process of iron degradation, it can also produce reactive oxygen and free radicals, resulting in cell and tissue damage. For this, some researchers do not consider it as a safe metal [54]. 

## 3. Tissue Reaction to the Metal Ion Toxicity

During the implantation of a biomaterial, there is damage to the adjacent tissues and, if the surfaces of the trauma do not converge, healing is slow with consequent discontinuity of the tissues with consequent formation of disfiguring scars or infections. The healing process begins with the granulation tissue formation [55]. This happens because it is an immune response (therefore not microbial), leading to the formation of a reactive granuloma (or foreign body granuloma) [56]. This depends on the shape, size, surface/volume composition ratio, porosity, and rate of implant degradation. This granuloma is constituted of multinucleated giant cells, which result from the aggregation of macrophages to engulf the implant and from granulation tissue (macrophage cells, sprouting new capillaries (angiogenesis), and abundant fibroblasts) [57]. A greater number of macrophages and multinucleated giant cells accumulate around implants with a rough surface (such as porous or intertwined except for those that are inserted into the bone where the osseointegration process occurs) and with a high surface-to-volume ratio compared to smooth surface implants. Therefore, the granulation tissue is a vascularized connective tissue. Angiogenesis begins 48–72 h after tissue injury and lasts several days. The granulation tissue formation follows inflammation and practically marks the onset of healing with the production of myofibroblasts and smooth muscle cells, which migrate to the wound within 3 days after implantation [58]. Thanks to the growth factors (TGFβ, PDGF, and FGF) secreted by macrophages, the fibroblasts can be multiplied and lead to the production of components of the extracellular matrix such as collagen type I and type III, proteoglycans, and fibronectin, which chemotactically act on them. Type I collagen is one of the components of the fibrous capsule, which surrounds the biomaterial when the healing process is complete. The type and progression of healing depend on both local (such as infection, blood perfusion, and more) and systemic (such as infections, pre-existing pathological conditions, and more) factors [59]. 

## 4. Oral Environment Bio-Mechanisms Reactions and Corrosion 

### 4.1. Chemical Mechanisms on the Surface of the Biomaterial (Cell-Surface Reactions)

**Figure 3:**
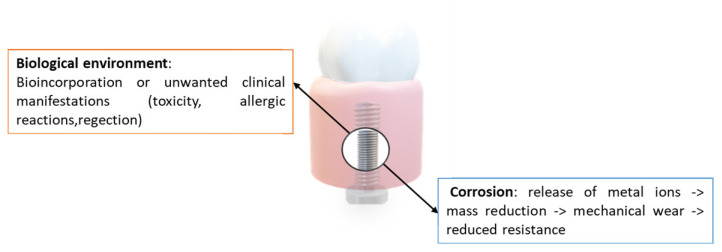
Biomaterial and biochemical reaction mechanisms: interaction between electrochemical metal reactions and biological environment. Therefore, the first critical factor in the evolution of the phenomenon from a material point of view is its surface condition. The consequences of electrochemical corrosion.

When a metallic biomaterial is inserted in a biological environment such as the oral cavity, an interaction occurs between the two materials (tissue—metal). Hence, biochemical reactions occur because there is the integration of the biomaterial and its long-term stay in the body. The metal contact surface in continuous transformation and evolution and if corroded by this environment, will progressively lose its mass integrity and consequently its mechanical strength (Figure 3) [60,61]. 

Once the implant is placed in the appropriate tissue, it meets body fluids at the implant site. Thanks to the predominant presence of water in each biological structure, it will be the first to meet the surface of the biomaterial [62]. Therefore, thanks to its polarity, the connection occurs with electronegative atoms of O_2_, while in polymers or composite materials with hydrogen atoms and oxygen or nitrogen groups that form hydrogen bonds. Hence, on the surface of the hydrated biomaterial, proteins that are linked by hydrogen bonds (such as fibronectin, albumin, IgG antibodies, factor C3, etc.) can be produced with the hydrogen atoms of the water molecules and gradually replace its water with the connection of biological molecular tissue with the metal [63,64]. Finally, the protein bonds form a network to the metal implant thus forming a winding that takes shape around the implant. The type and density of the proteins depend on the biomaterial and microenvironment of the implantation site. When proteins bind to the biomaterial, their enthalpy changes due to the damage to the protein structure (the α-helix conformation changes) thus losing the secondary structural conformation [63]. This condition leads to a sequence of incorrect biochemical stimuli also linked to the biomaterial itself. It has been noted that the formation of amyloid-like proteins causes irreversible damage and binds the mechanisms of fibril formation [65,66]. In addition, metal ions bind directly or indirectly to O_2_, N, and S atoms or to negative ions in the body. They can be one of the causes of corruption from the fact that these belong to transition metals, which according to the reactions of Fenton and Haber—Weiss are present in one of the phases of the deterioration of materials [66,67]. Hence, biomaterials that contain metal ions such as Fe, Ni, Ti, or Cu can initiate tissue damage. The production of hydroxyl radicals (HO) [68] contribute to a series of electron transfers and oxidation reactions that cause tissue damage around the implant site and also contribute to the maintenance of the patient’s inflammation, which is not of microbial origin but can lead to chronic disease. Furthermore, these two reactions prevail in the organism under the normal physiological conditions, where the transfer of electrical charges is enhanced by enzymes and metal ions occupy much wider oxidation states [69,70]. 

Subsequently, when the lymphocytes reach the surface of the biomaterial, they encounter the protein web that surrounds the biomaterial. Its adhesion and recognition occur by binding the proteins of the network to the specific adhesion proteins of the inflammatory cell membrane. This mechanism creates a dynamic local condition (therefore it does not remain stable), creating an equilibrium and an exchange (Vroman effect) [71]. The oxidative stress that occurs in the biological microenvironment of the implant can alter the physiology of proteins. Therefore, during the oxidative stress that develops during metabolism, the production of hydroxyl free radicals occurs and is more present in the micro-environment around the implant. Furthermore, oxidative stress is one of the causes of inflammation [72]. Hence, as we mentioned, hydroperoxyl oxygen anions, which are produced in large numbers during oxidative stress, play an important role in the corrosion initiation process. These anions easily transport electrons and thus the metal ions produced by the reaction are deoxidized according to the Fenton equation producing hydroxyl free radicals. Macrophages and giant cells remain at the implantation site throughout the in vivo residence and function of the implant (>20 years) [73]. The long-term presence and oxidative activity of macrophages, which release oxygen free radicals, in the implant area can cause gradual wear, aging, and corrosion of the implant resulting in late failure. The healing process around an implant is usually completed by the fibrous connective tissue capsule formation, which encloses and isolates it from the body [74]. 

Tissue injury at the implant site causes another set of different problems that arise from the nature of the material, biocompatibility, decay of the material, and the patient, which in some cases can cause aseptic loosening of the prosthesis. Thus, several factors lead to corrosion of biomaterials in addition to technical defects such as the use of ionizing radiation for metal sterilization of biomaterials [68,75]. During irradiation, the metal is ionized and the production of electrons leads to the acceleration of the corrosion phenomenon [76]. Therefore, specific reactions in the contact surface contribute to the onset of corrosion but also to the development of inflammation. On the other hand, metal ions migrate and can occupy sites of other metals in the biological environmental, disrupting the ionic homeostasis of cells. The body’s oxygen, as it is a free radical, can also accept electrons from the metal ions of the implants creating locally anoxic conditions, which stimulate various body enzymes by triggering a new free radical mechanism, subsequently causing oxidative stress, and thus maintaining in a state a chain wear reaction. Thus, oxidative stress develops during the placement of biomaterials leading to both the development of inflammation and direct redox reactions with implants [68]. The result of these conditions is the onset of corrosion, the type of which depends on the chemical composition of the alloys. Thus, the inflammation is primary or a continuation of acute inflammation and can last for weeks, months, or years. Conversely, if the presence of inflammation persists for a longer period, it is a strong indication of the establishment of an infection in the implant area. Inflammation is characterized by interactions between endothelial cells, monocytes, lymphocytes T, platelets, and the presence of chemotactic molecules (such as chemo-regular protein 1 and osteopontin modified LDL, which are produced by the peroxidation of proteins during oxidative stress and others) by increasing the degree of activation of these cells, induce hyperplasia cellular and strengthen the inflammatory reaction at the damage sites [77]. Chemotaxis molecules induce the expression of specific molecules (such as selectins, intercellular adhesion molecules, and others) on the cell surface for the adhesion of monocytes and T lymphocytes crossing the endothelial layer. The interactions of monocytes and T lymphocytes with chemotactic molecules [78]. 

The survival and proliferation of monocytes and T lymphocytes are ensured by factors such as the granulocyte-macrophage colony stimulating factor and interleukin-2, respectively. Inflammatory diseases (such as interferon-γ) appear to induce the apoptosis of macrophages and participate in the formation of the necrotic nucleus of complex lesions. Consequently, the immune response begins immediately after the foreign body enters the body. To prevent free radical reactions and the onset of corrosion and aging of the implants, coating materials have been developed with the presence of valid antioxidant compounds and anti-inflammatory properties [79,80,81]. 

### 4.2. Bio-Mechanisms of Erosion at the Implant Site 

**Figure 4:**
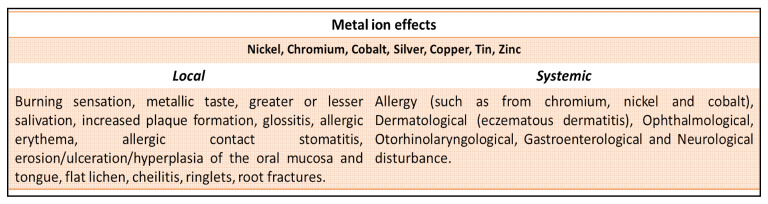
The figure indicates some symptoms that are induced by the ion currents mainly from Ni, chromium, cobalt, silver, copper, tin, and zinc and local effects caused by currents and the diffusion of metal ions.

As we have mentioned, in the implanted metallic biomaterial, some proteins are adsorbed on its surface. The proteins are derived from the blood stream and tissue fluids and then from cellular activity in the peri-implant area. The presence, locally, of an abundance of proteins and amino acids not only affects the biochemical process but also the erosion of the implant [68,82]. Macrophage-produced reactive oxygen derivatives corrode the metallic biomaterial during phagocytosis. Indeed, the adsorption of some proteins (i.e., IgM immunoglobulins), promote the activity of both polymorphonuclear neutrophils, monocytes, and even macrophages, which is the mechanism of opsonization. The largest cell population in the blood that are polymorphonuclear neutrophils are the first phagocytes to decant into the implantation site [83]. When no microbes or endotoxins are present at the implantation site, neutrophils are rapidly replaced by monocytes/macrophages within 12–24 h. Macrophages phagocytes have the advantage of longer survival and larger size, which allows them to engulf larger targets [84]. Metal is their primary target, so macrophages, in their attempt to engulf the metal biomaterial, increase the metabolic rate. For about 3 h after phagocytosis, significant oxygen consumption is observed, referred to as an oxidative or metabolic burst (respiratory or metabolic burst). They also increase their mobility and the production of enzymes and reactive oxygen products, but without effect, as phagocytosis cannot be completed [85]. In their attempt to increase their phagocytic capacity, macrophages unite and create giant cells and are the components of granulation tissue and promote the activity of fibroblasts by releasing cytokines. Fibroblasts create a connective tissue capsule that encloses the metallic biomaterial and isolates it from adjacent tissues. The thickness of the capsule depends on the micro mobility of the implant, the type of metal biomaterial, and the release rate of the metal ions [86]. It should be noted that the inclusion of the biomaterial by the connective tissue occurs in most part of the cases, but, in the case of titanium, osseointegration occurs, which is the “direct” structural and functional connection of the organized bone with the surface of a mechanically loaded implant. In the capsule of the connective tissue, metal ions originating from the corrosion of the metallic biomaterial are detected, because the conditions that prevail in the implantation area favor the phenomenon of corrosion [87]. At the beginning, in the implantation of a metallic biomaterial in the bone, the pH value drops to 5.2 for at least 2 weeks, after which it returns to the normal value of 7.4 and remains relatively constant thanks to the local buffer systems. The concentrations of chloride ions in plasma and connective tissue are 113 and 117 mEqlL-1, respectively. The Cl^−^ ions are responsible for the chemical disruption of the metallic biomaterials’ passive layer [68]. The metal ions released in the oral cavity spread to hard tissues (teeth, bones), soft tissues, saliva with subsequent diffusion into the digestive tract and blood with subsequent diffusion in the body, accumulation, and/or excretion. The local and systemic effects in the body correspond to the action of ion metals released (Figure 4) [72,88].

**Figure 5:**
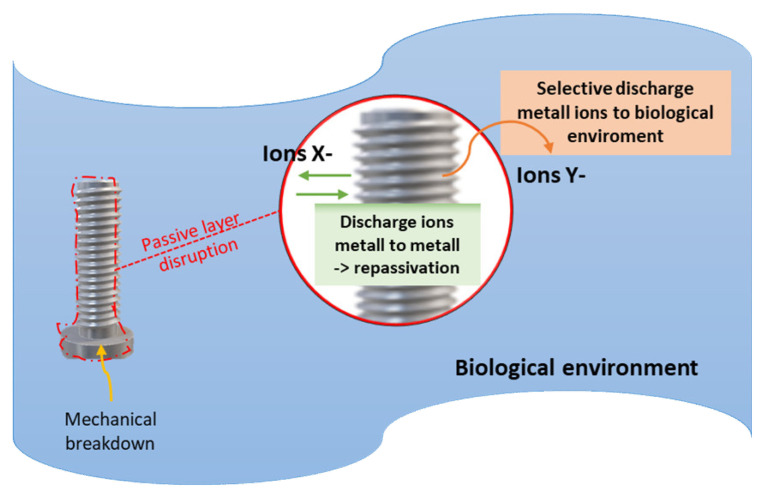
The employed passivate (both passivation and repassivation process). Breakthrough and repassivation of the passive layer [93]. The passive layer can be damaged by mechanical causes (mechanical breakdown), by abrasion or chemical decomposition, by the products of cellular metabolism, such as hydrogen peroxide and carbon dioxide, and also by the metal ions that the cell contains that can cause an exchange with the biological environment. The evolution of the phenomenon manifests itself in three moments: (i) the breakdown of the passive layer, (ii) the breakdown of the X^+^ and Y^+^ ions, and (iii) the X^+^ metal ions are released in the body, while the Y ^+^ restructure the layer. The X^+^ ions that are released in the implant site and, depending on the oxidative chemical characteristics, form organometallic compounds that are either removed or can replace the cellular metal ions causing biochemical changes [93,94].

The metallic biomaterial corrosion resistance depends on the passivation phenomenon that creates the passive layer on the surface of some alloys of a stable oxide and hydroxide layer with a thickness of less than 5nm on the surface of the metallic biomaterial and has a protective role—if in intact—thus contracting further corrosion [89,90]. The composition of the alloy significantly affects the structure and composition of the passive layer and thus the corrosion resistance of the alloy. The formation of this protective barrier between the metal and corrosive environment is mainly due to the electrochemical mechanism of O_2_. Furthermore, the low O_2_ concentration in the implant area is significantly reduced, which delays repassivation (Figure 5) [91,92]. 

### 4.3. Oral Microbiotas’ Influenced Metal Corrosion

After delivery, the germs colonized the newborn’s body (this will occur in any area of the body such as skin, oral cavity, nasopharynx, lung, intestine, system urogenital) from the first minutes of its life within 10–13 years old. Finally, with the first teeth, we will have new surfaces for microbial colonization and, subsequently, a change occurs after the replacement with the adult dentition. This oral colonization goes hand in hand with the gut microbiota which constitutes the great biological organ communicating with the other microbiota via cross-talking axes such as oral/lung [95].

Indeed, the oral microbiota is the one that most influences the lung microbiota, thanks to the translocation of microorganisms, such as “bio-aerosol” progression action, thus conveying them from the oral cavity to the lower airway’s tract [96]. The saliva is produced by the salivary glands and play a key role in the composition of the indigenous bacterial habitat’s communities because: (a) saliva is composed of organic substances (amino acids, mucoproteins, carbohydrates, vitamins, etc.) and minerals (Na, P, K, etc.), (b) as well as keeping the environment moist (mucous membranes and teeth are constantly immersed in saliva), has the properties of defenses against microorganisms through the action of antibacterial compounds proteins (such as lysozyme, cystinates, histamines, galactoxin, lactoperoxidase and calicidin, and others), which act on a salivary pH around 7 [97]. These antimicrobial components can affect the growth of certain microorganisms by limiting it. All microorganisms present in saliva are derived from the oral tissue micro composition by detaching themselves from their surface. However, the microbiota of saliva is mainly supplied by the bio-membrane of the tongue (mainly *Prevotella* and *Streptococcus* spp.) [98]. Several studies report that the oral microbiota of people in less well-off conditions are characterized by a lower biodiversity with the presence of bacteria at higher concentrations such as *Aggregatibacter Segnis*, *Achromobacter xylosoxidans*, and *Neisseria cluster II*, with the risk of periodontal and systemic diseases [95,99].

On the other hand, wealthy people have higher levels of *Rothia mucilaginosa, Megasphaera micronuciformis, Granulicatella adjacent, Veillonella parvula, Prevotella histicola, Fusobacterium periodontium*, and *Tannerella forsythia* [100,101]. In addition, the alterations increase processes such as the acidification of saliva and the decrease in oxygen, which leads to the development of anaerobic bacteria. Instead, bacteria such as *Nesseira subflava* and *Corynebacterium* are reduced in individuals who smoke. Antibiotics such as azithromycin, amoxicillin, clindamycin, and ciprofloxacin are antibiotics with the greatest impact on the microbiota leading to a decrease in *Actinomycetota phylum* [102,103,104].

Changes in bacterial functionality were also observed. Therefore, the oral microbiota eubiosis state is correlated to the pulmonary one, which in turn is linked to the intestinal one and consequently to all the axes of communication of the microbiota referred to above. As in oral cavities, the gut microbiota composition has a group of bacteria that helps to prevent the proliferation of some pathogenic bacteria. Indeed, *Bifidobacteria, Escherichia coli*, *Acidophilus influenzae*, *Enterococci* spp., and others inhibit the growth of those opportunistic bacteria, included laser therapy [105,106].

A metal material microbiologically influenced corrosion is due to microbial action through the development of a biofilm as well as the chemical composition of the metal surface. Biofilms are an organized community of microorganisms, bacteria, fungi, protozoa, which are found in the polysaccharides network, and are attached to a living or inactive surface [107,108,109].

Oral microbiota are highly prevalent in humans. Its evolution is strongly influenced by the *Streptococcus* spp., which are considered the main group of early settlers and predominates in the oral cavity. Oral microbiota biofilm can cause significant pitting corrosion and faster dissolution of metal ions including Ni and Cr. Hence, some electroactive microbiotas’ microorganisms via extracellular electron transfer from the oral microbiota may be the cause of accelerated corrosion [110]. *S. mutans* appear to be the main cause of caries. It synthesizes extracellular and intracellular polysaccharides from which it produces acids. It also produces bactericides that influence the formation of dental plaque [111]. They are divided according to antigens that have in eight different serotypes. After bacterial culture, the abundant oral commensal of the oral microbiota of *Streptococcus* spp. (such as *S. sanguinis* and *S. mutans*), the elution of Fe, Cr, and Ni from stainless steel orthodontic appliances but not in those produced by Ni/Ti material of these from oral bacteria releases metal ions (such as nickel), which can be one of the causes of allergic manifestations [112]. When, in the mouth, a metal corrodes (for chemical or biological reasons or for electrochemical corrosion), it sends ions into solution that diffuse in the saliva (then pass into the gastro-enteric tube) or are absorbed by the hard and soft tissues of the mouth and then pass into the bloodstream. The reactions that the organism opposes to this entry of ions (which are generally released in the form of insoluble salts) depend on the biochemical characteristics of the ion or of the salt itself [113]. Therefore, the metals that easily provide this type of salts (such as CuSO₄, AgCl, and others) cause, in a certain way, reactions that are toxic to the organism. Sulphur proteins (such as nickel and others) mainly cause allergic reactions. So, it can be said that the corrosion can be correlated by the “microbial digital import” that characterizes the microbiota, which thus causes it to be unique for everyone by the presence of a dysbiosis from food habits or toxic substances (such as abuse substances, heavy metals poisoning, etc.) and by the immunity related to it [114,115]. Indeed, an example is the biological corrosion of dental interest are those of orthodontic brackets in stainless steel and silver cones, which were formerly used as an occlusive material in endodontic treatments [112,113].

**Figure 6:**
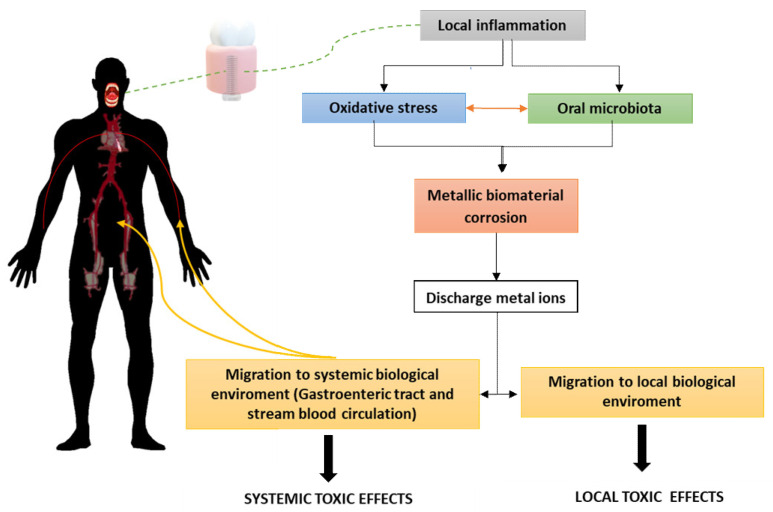
The main mechanisms of biomaterial corrosion that can lead to toxicological disorders.

A biofilm is an organized microbial community consisting of cells characterized by an irreversible adhesion to a substrate, surface, or between them, incorporation in a substrate of extracellular polymers (extracellular substances polymeric-EPS), which in themselves produce and have the appearance of a differentiated phenotype, in terms of the growth rate and gene expression [108,116]. The cells’ proximity conditions favor genetic differentiation (quorum sensing), with the consequent predominance of resistant forms [117]. The biological corrosion is caused by interactions, often synergistic, between the surface of the biomaterial, abiotic corrosion products, and bacteria with direct or indirect action (such as biofilm metabolites) [117]. Bacterial metabolites can be organic and inorganic acids or volatile compounds (such as ammonia and hydrogen sulfide). Indeed, the aluminum alloys structures can undergo corrosion from these metabolites, which for the most part is due to fungi rather than bacteria. The correction decreases as the molecular weight of the acid increases. The metabolites consist of various organic acids such as formic, succinic, citric, isocitric, aconitic, and 2-*oxoglutaric*, whereas enzymes associated with certain bacteria would preferentially attack the zinc and magnesium present in aluminum alloys [97,98,99,100,101,102,103,104,105,106,107,108,109,110,111,112,113,114,115,116,117,118,119]. Some bacteria can then reduce the sulphates or iron or produce. The most common forms of corrosion are therefore pitting, crevice corrosion, and stress corrosion cracking. In an oral environment coexist microorganisms that reduce sulphates (bacteria that reduce sulphates), i.e., *Bacteroides corrodens*, sulphur oxidizing bacteria such as *Thiotrix*, and acid-producing bacteria such as *S. Mutans*. These microorganisms form biofilms, which appear to be a strong risk factor for the biological corrosion of dental alloys together with the electrochemical reactions (Figure 6) [61,90,107,120].

Finally, this change that may exist in the oral microbiota, could be linked to certain systemic diseases. In fact, *S. mutans*, *Porphyromonas gingivalis*, and *Gemella haemolysans* are involved in several pathologies [121]. It has been noted that *S. mutans*, as well as being responsible for dental caries, contributes by altering the functionality of epithelial cells and thus facilitates the development of atherosclerosis, an important factor for the hypertensive state. Besides, *Porphyromona gingivalis* promotes the secretion of inflammatory cytokines following tissues that compromise and aggravate the condition of atherosclerosis by covering the atheromatous plaque and contribute to the development of coronary heart disease [122]. Additionally, other species leading to the development of oral microbiota dysbiosis with a possible subsequent impact in metal-based biomaterials are *Porphyromonas endodontalis*, *Campylobacter rectus*, *Prevotella intermedia*, *Prevotella nigrescens*, and *A. actinomycetemcomitans* [123]. Further focused research studies on this area between metal-based biomaterials and other qualitative and qualitative oral microbial reactions in correlation with systemic diseases will be useful in advancing knowledge for targeted therapeutic alternatives, i.e., the use of probiotics for rebalancing dysbiosis [124,125,126,127,128].

## 5. Conclusions

The metallic biomaterials used up to now are not inactive and, in contact with the biological environment, undergo corrosion thanks to oral microbiota and the electrochemical factors that can lead to a destruction of the metal biomaterial and cause toxic effects. The hydroxyl radical (HO) production contributes to a series of electron transfers and oxidation reactions that cause tissue damage around the implant site and contribute to the maintenance of the patient’s inflammation, which is not of microbial origin but can lead to chronic disease. The phenomenon of biological corrosion is multifactorial and still under study. It depends on the body temperature, the oxygen of the implantation area, the pH of the surrounding biological fluid, the vital ions (Na^+^, K^+^, Ca ^2+^, Mg^2+^, Cl^−^, HCO_3_^−^, and HPO_4_^2−^) macrophage cells, etc. We can say that metals form inorganic salts (such as AgCl and CuSO_4_) that cause toxic-type reactions, the metals that produce organic compounds more easily (Ni, Au, and other linked to sulfur proteins) mainly cause allergic-type reactions. For this, it seems that the biochemical reactions of the organism are of such a complexity as not to allow for great simplifications: consider that about three thousand different enzymes have so far been found, of which about a third use a metal as a catalyst, and that there is nothing to suggest that the search for enzymes is at an end. Furthermore, it should be remembered that the same metal can cause different reactions depending on the type of compound it has formed and, therefore, on its biological availability.

## Data Availability

Not applicable.
